# Treatment of Diabetic Foot with Autologous Stem Cells: A Meta-Analysis of Randomized Studies

**DOI:** 10.1155/2020/6748530

**Published:** 2020-07-16

**Authors:** Jiezhi Dai, Chaoyin Jiang, Hua Chen, Yimin Chai

**Affiliations:** Department of Orthopedic Surgery, Shanghai Jiao Tong University Affiliated Sixth People's Hospital, Shanghai, China

## Abstract

**Background:**

This meta-analysis was to evaluate the efficacy of autologous stem cell administration for the treatment of diabetic foot.

**Methods:**

The electronic databases included PubMed, EMBASE, BIOSIS, Cochrane central, and Google Scholar internet, last updated on May 30, 2019. Evaluated outcomes included the rate of wound healing and amputation. Dichotomous outcomes were described as risk ratios (RR) with 95% confidence intervals (CIs). Statistical analysis was performed with RevMan 5.0 software and STATA 10.0 software.

**Results:**

Eight randomized controlled trial (RCT) studies were included in this study. The meta-analysis showed a lower amputation (RR 0.25, 95% CI 0.11 to 0.54, *I*^2^ = 0) and a higher wound healing rate (RR 2.05, 95% CI 1.67 to 2.51, *I*^2^ = 4) in the cell therapy group compared with control.

**Conclusion:**

This meta-analysis supports the effective role of stem cell therapy in promoting wound healing and decreasing rate of amputation in diabetic foot. In the future, more high quality and well-designed studies are need.

## 1. Introduction

Diabetic foot, a serious complication in type 2 diabetes, afflicts approximately 6% of people with diabetes worldwide [1]. It is associated with peripheral neuropathy or peripheral artery disease which increases risk of impaired wound healing and is the most important precursor for lower-extremity amputations [2, 3]. The resulting high number of amputations has major influence on the quality of life and constitutes serious clinical issue.

In recent years, a large number of clinical trials have shown a positive effect of new treatment modality using regenerative potential of the autologous stem cells transplantation [4]. In 2017, a meta-analysis by Guo et al. reported the effect of autologous stem cell administration in the treatment of diabetic foot ulcer [5]. The authors stated that stem cell administration was significantly favorable for healing diabetic ulcers.

To illustrate the need for regular updates in meta-analysis, we conducted a cumulative meta-analysis. More clinical trials were included in this study. We attempted to assess the efficacy of autologous stem cell administration for the treatment of diabetic foot.

## 2. Methods

The systematic review and meta-analysis was performed according to the Preferred Reporting Items for Systematic Review and Meta-Analyses (PRISMA) guidelines [6].

### 2.1. Study Selection

The electronic databases included PubMed, EMBASE, BIOSIS, Cochrane central, and Google Scholar internet. The final search was updated on May 30, 2019. There were no restrictions as regards the language. We reviewed the bibliographies of original trials, gray literatures, and review articles identified for potential eligible articles. The search terms included “stem cell(s),” “bone marrow,” or “cell therapy” paired with “diabetic,” and “diabetes” paired with “wound,” “ulcer,” “foot,” or “ischemia.” The search strategy was designed and refined, and two investigators conducted the search strategy to select references. In case of disagreement, it was discussed and consulted by a senior investigator.

### 2.2. Data Extraction

Two investigators independently extracted all relevant data. Disagreement was resolved with discussion and with adjudication by a third investigator if needed. Effective data included basic information (author name, publication year, study design, country, sample size, and follow-up), patient demographics (age and sex), intervention (type and dose of stem cell), and outcomes (rate of wound healing and amputation).

### 2.3. Inclusion and Exclusion Criteria

The inclusion criteria included [1] randomized controlled trials (RCTs); [2] skeletally mature patients, aged 18 or older with diabetic foot; [3] patients treated with autologous stem cell defined as the treatment group and participants in the control treatment arm who had conventional conservative therapy and/or administration of an inert placebo such as isotonic saline; and [4] outcomes that included the rate of wound healing and amputation. The exclusion criteria included [1] studies lacking measurement data and [2] animal models.

Diabetic foot disease is defined as “infection, ulceration or destruction of tissues of the foot associated with neuropathy and/or peripheral artery disease in the lower extremity of people with diabetes” [7].

Conventional therapy included adjustment of blood glucose, blood pressure and blood lipids, debridement to remove extensive callus and necrotic tissue, pressure-relief after wound dressing, and application of antibiotics.

The rate of wound healing was defined as the percent of patients whose wounds were healed at a given time point (wound size of 0 cm and Wagner score of 0 for each wound). The rate of amputation was defined as the percent of patients with the removal of the limb or a part of it above the ankle at a given time point.

### 2.4. Statistical Analysis

The study was performed with the Cochrane Collaboration's RevMan 5.0 software. For dichotomous data (amputation rate and wound healing rate), we used risk ratio (RR) with 95% confidence intervals (CIs) to measure outcomes. Heterogeneity among studies was assessed by the *I*^2^ statistic (with *I*^2^ > 50% indicating high heterogeneity) and chi-square tests (with *P* < 0.05 representing heterogeneity). A random effects model analysis was used as significant heterogeneity indicated.

Publication bias was evaluated with Begg's rank correlation test [8] and Egger's regression test [9]. Funnel plots were also used to test for publication bias. Data were tested with STATA 10.0 software. *P* < 0.05 indicated statistical significance.

### 2.5. Assessment of Methodological Quality

The methodological quality of RCTs was assessed with the Cochrane Collaboration's tool [10]. Five main fields included sequence generation, allocation concealment, binding, incomplete outcome data, and selective outcome reporting. For each item, studies were categorized as high, low, or unclear risk of bias.

## 3. Results

**Figure 1:**
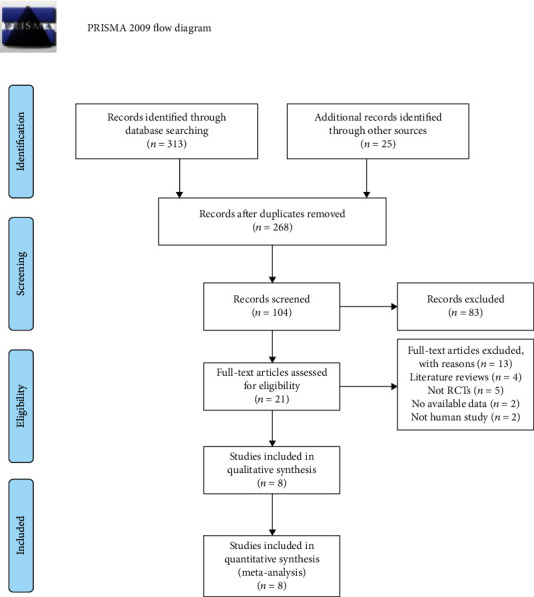
Flow chart of study selection in the systematic review.

A total of 268 articles were identified with the use of our search strategy, and the process of study selection is shown in Figure 1. Finally, eight RCTs involving 348 patients and 367 limbs were included in our study [11–18]. Three articles were published in China [11–13], one was from India [14], one was from Turkey [15], one was from Iran [16], one was from Korea [17], and one was from Germany [18]. Stem cells, including peripheral blood mononuclear cells (PBMCs), bone marrow mesenchymal stem cells (BMMSCs), bone marrow mononuclear cells (BMMNCs), human processed lipoaspirate (PLA), and bone marrow-enriched tissue repair cells (BMTRCs), were transplanted by intramuscular injection. The study of Lu et al. was divided into two groups treated with BMMSCs or BMMNs, respectively, and the study of Kirana et al. was divided into two groups treated with BMMNCs or BMTRCs, respectively. Follow-up ranged from two to thirteen months. Details of included trials are documented in Table 1.

The quality of included studies is shown in Table 2.

**Figure 2:**
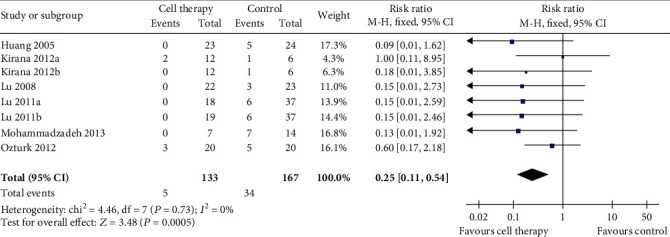
Forest plot showing the effect of stem cell therapy on amputation rate.

Six studies reported the outcome of amputation rate (Figure 2). The meta-analysis showed a lower amputation in the cell therapy group compared with the control group (RR 0.25, 95% CI 0.11 to 0.54, *I*^2^ = 0). Results gave a pooled rate of 3.76% (5/133) in the cell therapy group and of 20.36% (34/167) in the control.

**Figure 3:**
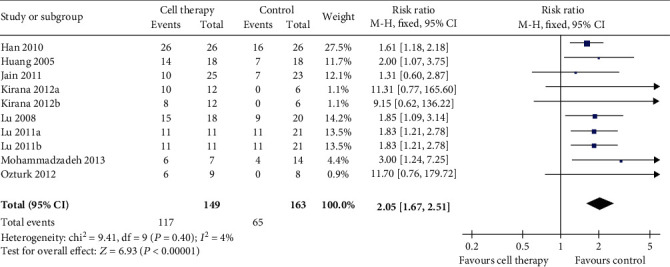
Forest plot showing the effect of stem cell therapy on wound healing rate.

Eight trials reported the result of wound healing rate (Figure 3). The meta-analysis showed a higher wound healing rate in the cell therapy group compared with the control (RR 2.05, 95% CI 1.67 to 2.51, *I*^2^ = 4). Results gave a pooled rate of 78.52% (117/149) in the cell therapy group and of 39.88% (65/163) in the control.

**Figure 4:**
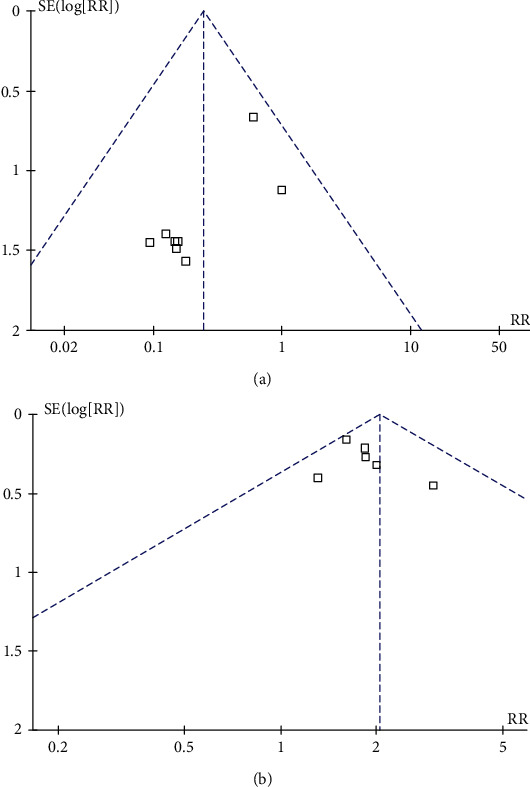
Publication bias in relation to amputation rate (a) and wound healing rate (b).

The funnel plot and statistical test showed publication bias in wound healing rate and no publication bias in amputation rate (Figure 4). Begg's test (*P* = 0.386) and Egger's test (*P* = 0.244) was in relation to risk of amputation rate. Begg's test (*P* = 0.002) and Egger's test (*P* = 0.03) was in relation to risk of wound healing rate.

## 4. Discussion

Diabetic foot typically presents as ulcers, infection, or destruction of tissues of the foot [19]. Conventional therapy of diabetic foot fails in 25% of patients and leads to amputation, which impairs patients' quality of life and affects social participation and livelihood [2]. Autologous stem cell therapy is gradually known as a new therapy. In this study, autologous implantation of stem cells improved ulcer healing rate and reduced amputation rate. Stem cell therapy may alter the outcome of diabetic foot to a certain degree.

Recent advances in stem cell research in both human and experimental animals have shed some light for clinical application of diabetic foot [20]. Diverse sources and the potential of self-renewing and multidifferentiation are main characteristics of stem cells [21]. BMMSC transplantation has been reported to improve cardiac function [22] or limb ischemia [23]. Xu and Liang reported that autologous PBMC transplantation can promote the establishment of collateral circulation in patients with a diabetic foot [24]. BMMNCs were reported to be more effective in the healing of foot ulcers compared with repeated percutaneous transluminal angioplasty [25]. Studies of different designs cannot be assessed in unification. Thus, we performed this meta-analysis to evaluate the efficacy of stem cell therapy for diabetic foot.

Previous meta-analysis on the problem has been discussed. According to the work of Guo et al. [5], stem cell administration has certain advantages for diabetic ulcers healing. Four studies were included in the study. It was limited by small sample sizes with poor quality. We included four extra trials that did fulfill our strict inclusion criteria and performed a new meta-analysis. We not only assessed the role of autologous stem cell administration on the diabetic wound healing but also evaluated the association of the treatment with amputation rate. The results reported by Guo et al. [5] were similar to ours; besides, there was a significant reduction in amputation rate after autologous stem cell treatment (RR 0.25, 95% CI 0.11 to 0.54).

Stem cell therapy may involve a variety of cell types. BM-MSC, PB-MSC, hUC-MSC, and ADSC were the most frequently used stem cell types in clinical application [26]. In this meta-analysis, BMMSCs and PBMCs were the most frequently used cell types in involved studies (*n* = 3). BMMNCs were used in two studies. PLA and BMTRCs were used in each study. All the cell types used have many advantages such as donor-specific therapy, lower malignancy risk, cell lineage committed (targeting differentiation), and no ethical conflict [26]. The best stem cell type to diabetic foot treatment remains controversial. Bone marrow was used as the chief source for stem cell therapy in clinical and preclinical studies, such as BMMSCs and BMMNCs. Lu et al. found the infusion of BMMSCs was more effective than that of BMMNCs in increasing lower limb perfusion [13]. PBMCs were used more frequently in clinical researches than in preclinical researches. Fadilah et al. found mobilized PBSCs are more preferred over bone marrow stem cells because of relative ease of collection and avoidance of anaesthesia and pain associated with bone marrow biopsy [27]. To assess the optimal type of stem cell, more high-quality and well-designed studies are needed in the future.

Although eight RCTs assessed the role of cell therapy in this meta-analysis, the heterogeneity among these studies weakened the strength of evidence. Stem cell sources, the number of stem cells, and routes of implantation differed among the trials. Therefore, future clinical studies with comparable protocols, doses, cell types, and administration routes are required to allow good comparison of these expected studies.

Potential limitations were reported in our study. Firstly, we included eight studies involving 338 patients. It showed the quantity of researches was small. Secondly, only English articles with positive results were included, which may cause publication bias. Finally, the role of cell therapy on major limb salvage should be discussed. We need more information to further differentiate major amputation (any resection proximal of the ankle) and minor amputation (any resection through or distal of the articulation of the ankle) [28].

In conclusion, this meta-analysis supports the effective role of stem cell therapy in promoting wound healing and decreasing rate of amputation in a diabetic foot. In the future, more high-quality and well-designed studies are needed. Standardization in the transplantation method, stem cell source, and quantity should be valued in future application [29].

## Figures and Tables

**Table 1:** Characteristics of the included studies.

	Country	No. of patients		Average age		No. of male		Treatment strategy		Follow-up (months)
		Treatment	Control	Treatment	Control	Treatment	Control	Treatment	Control	
Huang 2005	China	14^a^	14^b^	71.1	70.9	9	9	PBMCs (3∗10^9^)+C	Prostaglandin E1+C	3
Lu 2008	China	25	25	66.6	65.5	11	15	BMMSCs (7.32∗10^8^-5.61∗10^9^)+C	C	3
Lu 2011	China	20	41	63	64	—	—	BMMSCs (9.3∗10^8^)+C	Normal saline+C	6
		21		65		—		BMMNCs (9.6∗10^8^)+C		
Jain 2011	India	25	23	54	58	17	15	BMMSCs+C	Peripheral blood+C	3
Ozturk 2012	Turkey	20	20	79.9	70.8	16	13	PBMCs (9.92∗10^8^-1.24∗10^9^)+C	C	3
Mohammadzadeh 2013	Iran	7	14	63.5	64.2	—	—	PBMCs (0.9∗10^9^-1.2∗10^9^)+C	PBS+C	12
Han 2010	Korea	26	23	66.5	68.4	15	14	PLA (4∗10^6^-8∗10^6^)+C	C	2
Kirana 2012	Germany	12	6	68.5	—	9	—	BMMNCs (3∗10^8^)+C	C	13
		12		70.9		10		BMTRCs (0.8∗10^8^)+C		

PBMC: peripheral blood mononuclear cells; BMMSCs: bone marrow mesenchymal stem cells; BMMNCs: bone marrow mononuclear cells; PLA: Human processed lipoaspirate; BMTRCs: bone marrow-enriched tissue repair cells; C: conventional therapy. ^a^14 patients with 23 limbs; ^b^14 patients with 24 limbs.

**Table 2:** Assessments of risk of bias of the randomized controlled trials.

Studies	Sequence generation	Allocation concealment	Blinding	Incomplete outcome data	Selective outcome reporting
Huang 2005	Unclear risk	High risk	High risk	High risk	Low risk
Lu 2008	Unclear risk	High risk	High risk	High risk	Low risk
Lu 2011	Low risk	Unclear risk	Low risk	Low risk	High risk
Jain 2011	Low risk	Low risk	High risk	High risk	Low risk
Ozturk 2012	Low risk	High risk	High risk	High risk	Low risk
Mohammadzadeh 2013	Unclear risk	High risk	High risk	High risk	Low risk
Han 2010	Low risk	Low risk	Unclear risk	Low risk	Low risk
Kirana 2012	Low risk	Low risk	Unclear risk	Low risk	Low risk

## Data Availability

The data used to support the findings of this study are available from the corresponding author upon request.

## Abbreviations

PRISMA: Preferred Reporting Items for Systematic Review and Meta-Analyses
PBMCs: Peripheral blood mononuclear cells
BMMSCs: Bone marrow mesenchymal stem cells
BMMNCs: Bone marrow mononuclear cells
PLA: Human processed lipoaspirate
BMTRCs: Bone marrow-enriched tissue repair cells
CI: Confidence interval
RR: Risk ratios
SMD: Standard mean differences
RCT: Randomized controlled trial.
